# OPG*/*RANK*/*RANKL signaling axis in patients with type I diabetes: Associations with parathormone and vitamin D

**DOI:** 10.1186/s13052-019-0748-1

**Published:** 2019-12-10

**Authors:** Paraskevi Karalazou, Dimitrios Ntelios, Fani Chatzopoulou, Aikaterini Fragou, Maria Taousani, Konstantina Mouzaki, Assimina Galli-Tsinopoulou, Sofia Kouidou, Georgios Tzimagiorgis

**Affiliations:** 1 Laboratory of Biological Chemistry, Medical School, Faculty of Health Sciences, Aristotle University of Thessaloniki, 541 24 Thessaloniki, Greece; 2 4th Department of Pediatrics, Medical School, Faculty of Health Sciences, Aristotle University of Thessaloniki, Papageorgiou General Hospital, Thessaloniki, Greece

**Keywords:** Type 1 diabetes mellitus, Osteoprotegerin, Receptor activator of nuclear factor kappa-B ligand, Receptor activator of nuclear factor kappa-B

## Abstract

**Background:**

Type 1 diabetes (T1D) has been associated with a higher fracture risk due to alterations in bone structure and metabolism. On the other hand, the important role of the RANKL/OPG/RANK signaling axis in bone physiology is well established. The aim of this study was to evaluate the levels of receptor activator of nuclear factor kappa-B ligand (RANKL), receptor activator of nuclear factor kappa-B (RANK) and plasma osteoprotegerin (OPG) levels, in T1D youngsters and to investigate factors that could influence the *OPG*/*RANK*/*RANKL* signaling axis such as 25-hydroxy vitamin D [25(OH) D], parathormone (PTH) and age.

**Methods:**

Serum RANKL, RANK, 25(OH) D, PTH levels and plasma OPG levels, were measured in 71 youngsters with T1D and 50 healthy controls matched for age and gender.

**Results:**

Plasma OPG levels were significantly lower (*p* = 0.025) in T1D patients compared to controls. Serum RANKL levels were significantly higher (*p* = 0.037), while no differences were observed in serum RANK levels (*p* = 0.946) between the two groups. Serum 25(OH) D levels found significantly decreased (*p* < 0.001) while serum PTH levels were significantly elevated (p < 0.001) in T1D patients than in controls.

**Conclusions:**

Our results demonstrated that OPG and RANKL may be promising biomarkers for T1D patients. However, their circulating levels were associated with several factors including PTH, 25(OH) D and therefore, may represent an integrative biomarker for a variety of endocrine signaling disturbances observed in T1D.

## Background

Type I diabetes (T1D), a common chronic childhood disease, is characterized by insulin deficiency caused by the progressive destruction of pancreatic beta-cells *by autoimmune* processes [1]. T1D is *usually* first *diagnosed* in childhood or early adulthood and its incidence has been constantly increasing worldwide [2–4]. Along with the well-known microvascular and macrovascular complications, T1D affects bone metabolism. In particular, there is evidence for poor bone mineralization, abnormal glycosylation and cross-linking of skeletal collagen [5–7]. Consequently, this reduced bone strength and quality can lead to fractures at any site, predominantly at femoral neck [7, 8].

There is a great need to improve our understanding of the mechanisms behind the development of bone complications in diabetic patients and to develop new biomarkers that better stratify high-risk patient groups in a preclinical stage. To this extent, the study of OPG/RANKL/RANK signaling axis in diabetes has been proposed as a promising new research direction. Bone remodeling depends on the balance between bone formation performed by osteoblasts and bone resorption induced by osteoclasts. Previous studies have identified the *OPG*/*RANK*/*RANKL* signaling axis, among others, as an important regulator of this process [9]. In particular, OPG is a soluble glycoprotein produced mainly by osteoblasts that inhibits osteoclastogenesis by preventing the binding of RANKL to its receptor RANK [10]. On the other hand, RANKL is an osteoclast differentiation factor produced by osteoblasts which triggers osteoclastogenesis by binding to RANK, a membrane receptor expressed by osteoclast precursors [11]. Therefore, RANKL and OPG are key factors in bone remodeling process that is carried out by osteoblast and osteoclast cells within the *bone* remodeling *unit* [12, 13].

So far, studies examining OPG and RANKL circulating levels have led to inconsistent results in *children and adolescents with diabetes* [14–18]. Therefore, the aim of this study was to test the hypothesis that circulating levels of RANKL, RANK and OPG are altered in patients with T1D compared to normal controls and to investigate factors that could significantly affect concentrations of OPG and RANKL such as vitamin D, parathyroid hormone and age.

## Subjects and methods

### Subjects

This case-control study included 71 children and adolescents with T1D and 50 healthy controls matched for age and sex (Table 1). Participants were recruited from January of 2015 until January of 2017. Entry criteria for the patients were age 5–18 years and duration of diabetes of more than 4 years. Entry criteria for controls were age 5–18 years and no medical history. Exclusion criteria for both patients and controls were the presence of chronic metabolic diseases that could affect bone metabolism, such as metabolic syndrome, hypothyroidism and other autoimmune diseases, renal disease, liver disease etc. In all T1D patients diabetic complications such as retinopathy, nephropathy, neuropathy and metabolic syndrome were excluded by frequent appropriate laboratory and clinical examinations. In order to detect early microalbuminuria a 24 h urine collection was performed and the urinary ratio albumin (A) to creatinine (C) was evaluated (normal values A/C less than 30 mg/24 h). Written, informed consent was obtained from all participants and the study was aproved by the appropriate institutional review board.

**Table 1:** Demographic data and measured biomarkers in T1DM patients and controls

	T1DM (*n* = 71)	Controls (*n* = 50)	*p* value
Gender (boys/girls)	41/30	26/24	0.580^a^
age (years)	13 (11–16)	12 (10–13.2)	0.071^b^
Duration of diabetes (years)	5 (4–8)	–	–
HbA1c (%)	7.5 (6.8–8.2)	5.3 (5.1–5.3)	< 0.001**^b^
Glu (mg/dl)	203 ± 73	86 ± 9	< 0.001**^c^
Ca (mg/dl)	9.75 ± 0.38	9.62 ± 0.36	0.066^c^
P (mg/dl)	4.39 ± 0.52	4.42 ± 0.9	0.862^c^
Mg (mg/dl)	1.92 (1.76–2.04)	1.95 (1.8–2.09)	0.105^b^
ALP (U/L)	222 ± 110	151 ± 82	< 0.001**^c^
PTH (pg/ml)	46.75 ± 17.1	26.56 ± 9.87	< 0.001**^c^
25(OH) D (ng/ml)	26.6 (21.8–32.3)	54.3 (48.7–60.5)	< 0.001**^b^
SGOT (U/L)	19 (15–24)	21 (17–27)	0.092^b^
SGPT (U/L)	15 (12–19)	14 (12–18)	0.514^b^
ACR ratio	3.35 (2.47–5.97)	–	–
OPG (pg/ml)	314 (171–504)	392 (271–667)	0.025*^b^
RANKL (pg/ml)	545 (107–3377)	234 (12–2046)	0.037*^b^
RANK (pg/ml)	43 (18–110)	52 (18–91)	0.946^b^
RANKL/OPG	1.7 (0.41–7.22)	0.64 (0.02–2.95)	0.002*^b^

Data are presented as mean ± standard deviation for normally distributed variables and median (interquartile range) for non normally distributed variables

*: significance *p* < 0.05, **: significance *p* < 0.001

^a^: Chi-square test for qualitative variables, ^b^: Mann-Whitney U test for not normally distributed quantitative variables. ^c^: t-test for normally distributed quantitative variables

### Biochemical measurements

Blood samples were collected in the morning, in a fasting state. The serum or plasma samples were frozen at -80 °C until testing. Also the supernatants of the 24 h urine samples were frozen.

Concentrations in serum of Glu, Ca, P, Mg, ALP, SGOT and SGPT as well microalbuminuria in a 24 h urine sample were measured using colorimetric biochemical assays in an Architect c800 Clinical Chemistry Analyzer. Intact (1–84) PTH and Vitamin D [25(OH) D] were measured in serum with ECLIA method in an Architect i1000SR Immunoassay Analyzer (Abbott Laboratories, Abbott Park, Illinois, U.S.A.), where their normal ranges were 14–72 pg/ml and 40–100 ng/ml, respectively and the inter-assay coefficients of variability were 8 and 10%, respectively. 25(OH) D insufficiency was set to < 30 ng/ml. Whole blood HbA1c levels were measured using HPLC method in a Menarini ARKRAY ADAMS™ A1C HA-8180 Analyzer (Menarini Diagnostics, Florence, Italy).

Serum RANKL and RANK concentrations were measured, applying Sandwich ELISA (BosterBio, Pleasanton, CA, USA). Human TNFSF11/RANKL ELISA Kit had a sensitivity of less than 0.43 pmol/l with an intra-assay precision of 5.1% and an inter-assay precision of 5.7%. Human receptor activator of NFkB, RANK ELISA Kit, had a sensitivity of less than 0.04 pmol/l with an intra-assay precision of 5.4% and an inter-assay precision of 6.6%. Plasma OPG concentrations were measured by a sandwich ELISA, applying a commercially available kit (Cohesion Biosciences, London, UK) with a detection limit less than 0.09 pmol/l, according to the manufacturer’s instructions. All samples were measured in duplicate and averaged.

### Statistical analysis

Statistical analysis was performed using a SPSS software version 25 (SPSS Inc., Chicago, IL, USA). Data were presented as mean ± standard deviation for normally distributed variables and median (interquartile range) for not normally distributed variables. Kolmogorov-Smirnov test was used for the assessment of normality. Chi-square-test was used for comparison of categorical variables. t-test and Mann-Whitney U test was used as appropriate to test for differences in continuous variables. Pearson’s correlation coefficient (r) and Spearman’s correlation coefficient (r_s_) were used for normally and not normally distributed variables respectively. Level of significance was set at *p* < 0.05.

## Results

### Circulating levels of OPG, RANKL, RANK in patients with Τ1D compared to controls

**Fig. 1:**
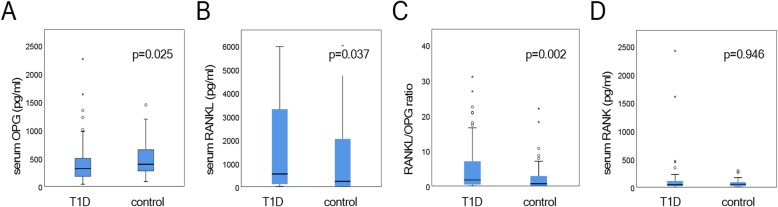
Plasma levels of OPG (**a**) RANKL (**b**) in T1D patients and controls. **c**. RANKL/OPG ratio in T1D patients and controls. **d** Serum levels of RANK in T1D patients and controls

OPG plasma levels were significantly lower in patients with T1D than controls (*p* = 0.025) (Fig. 1a). On the other hand, serum RANKL were significantly higher in the patients group (*p* = 0.037) (Fig. 1b). The RANKL/OPG ratio was also found to be significantly higher in the patients (*p* = 0.002, Fig. 1c). Interestingly, RANK, despite being a transmembrane protein, it was in detectable levels in the serum. However, serum RANK concentrations did not differ significantly between patients with T1D and controls (*p* = 0.946) (Fig. 1d). Interestingly, OPG, RANKL and RANK levels *were not associated* with *glycemic control* as measured by HBA1c (Table 2).

**Table 2:** Associations of bone markers with HBA1c

	Correlation coefficient	*P* value
OPG	0.112	0.354^*^
RANKL	−0.025	0.843^*^
RANK	0.05	0.705^*^
PTH	−0.117	0.339^**†**^
25(0H)D	−0.085	0.479^*^
ALP	−0.153	0.202^**†**^
Ca	−0.044	0.717^**†**^
P	−0.085	0.483^**†**^
Mg	−0.250	0.035^*§^

^*^ spearman correlation, ^**†**^ pearson correlation, ^§^ significant at *p* < 0.05

### 25(OH) D, PTH and age as critical factors influencing OPG and RANKL levels

**Fig. 2:**
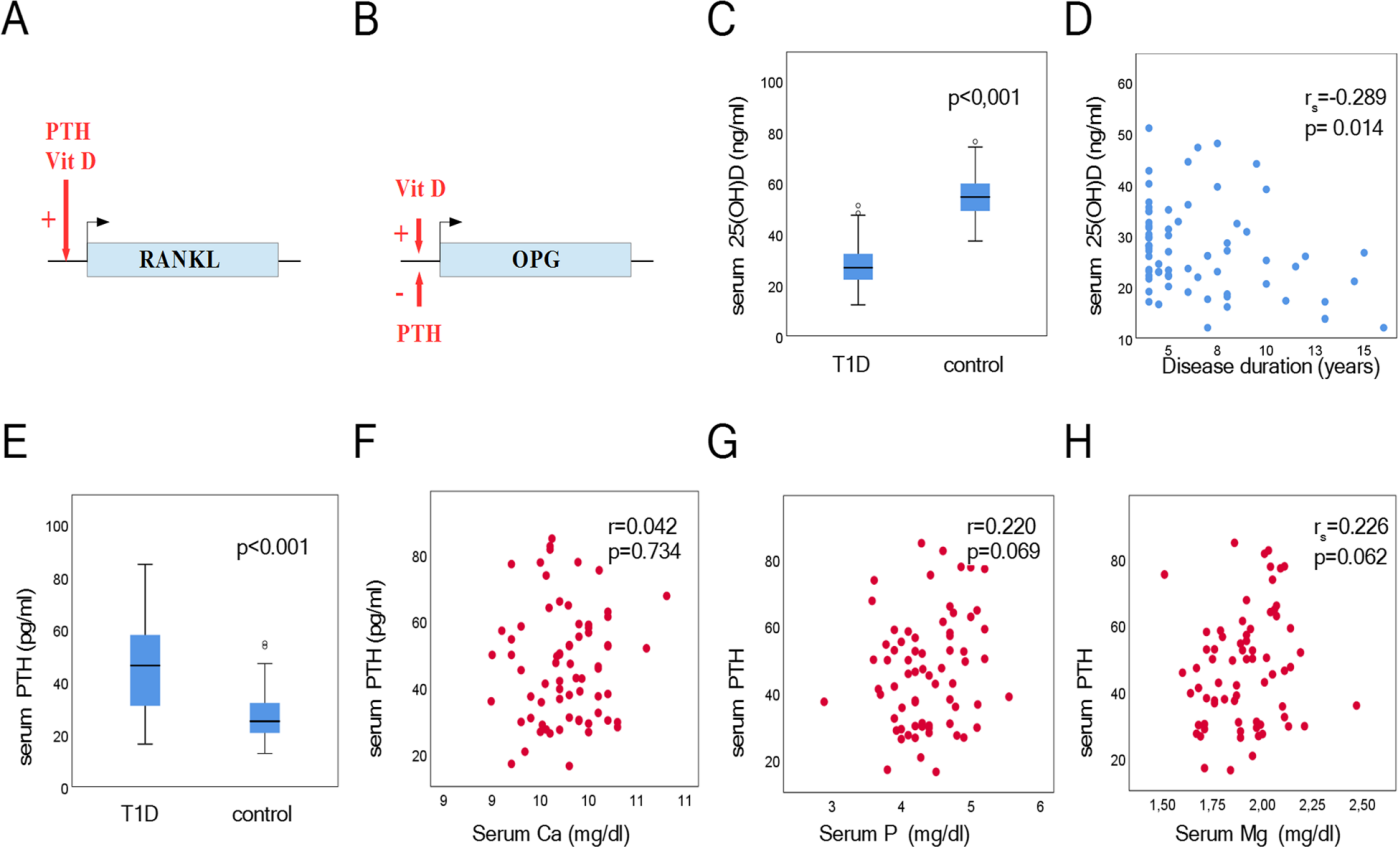
Regulation of OPG (**a**) and RANKL (**b**) expression. (**c**) Serum levels of 25(OH) D in T1D patients and controls. **d** Scatterplots depicting the relationship of serum 25(OH) D levels with disease duration. **e** Serum levels of PTH in T1D patients and controls. Scatterplots depicting the relationship of serum PTH levels with calcium (**f**), phosphorus (**g**) and magnesium levels (**h**) in T1D patients

**Fig. 3:**
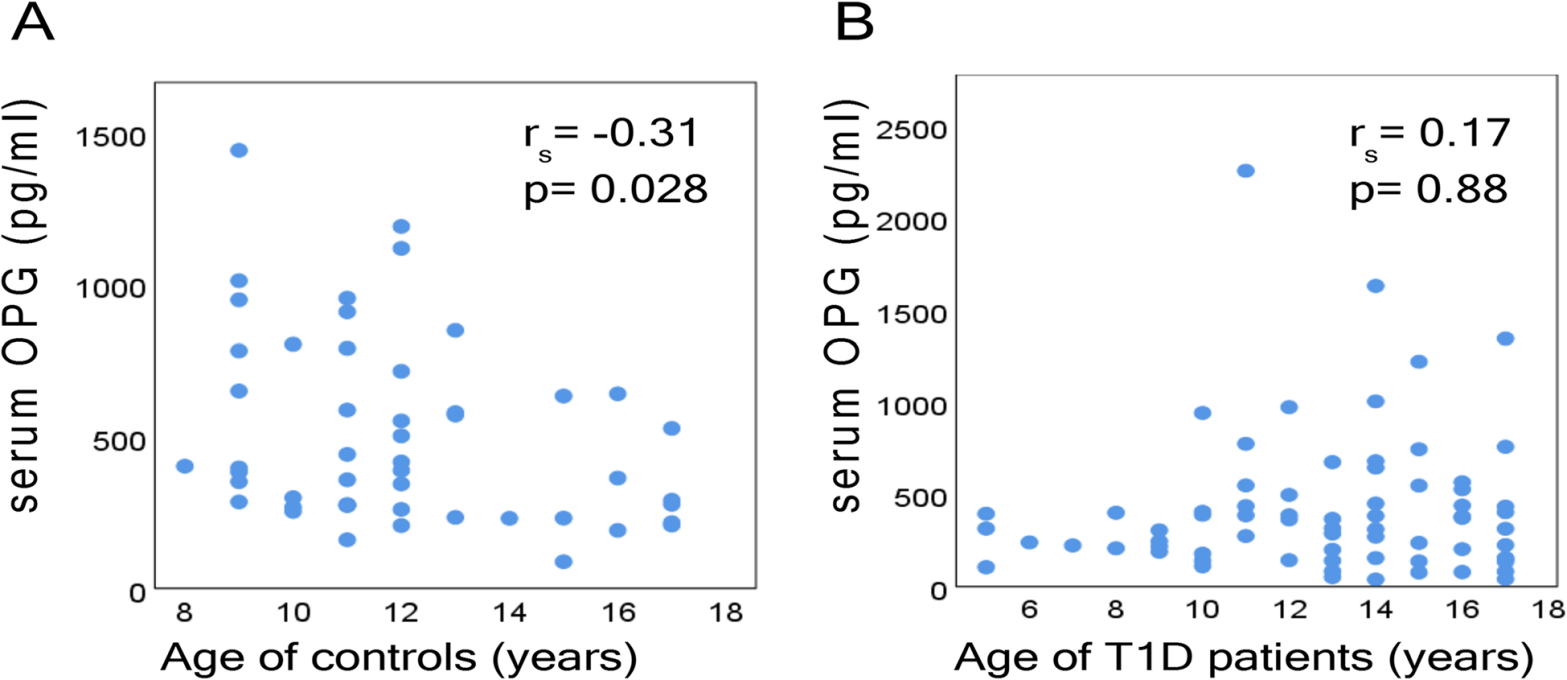
Scatterplots depicting the relationship of serum OPG levels with age in (**a**) controls and (**b**) T1D patients

Our results revealed that 25(OH) D serum levels were decreased significantly in the patient group (*p* < 0.001) (Fig. 2c) whereas PTH serum levels increased significantly (*p* < 0.001) (Fig. 2e). Interestingly, *longer disease duration* was *associated* with lower 25(OH) D levels (r_s_ = − 0.289, *p* = 0.014) (Fig. 2d). Furthermore, OPG levels correlate significantly with age in the control group (r_s_ = − 0.31, *p* = 0.028) (Fig. 3a). However, this association was not observed T1D patients (r_s_ = 0.17, *p* = 0.88) (Fig. 3b).

### Circulating bone metabolism markers in patients with Τ1D compared to controls

Serum Ca, P and Mg levels showed no statistically significant difference between patients and controls (*p* = 0.066, *p* = 0.862 and *p* = 0.105, respectively). Additionally, serum Ca, P and Mg levels were not associated with PTH levels (Fig. 2). Interestingly, serum ALP levels were significantly higher in T1D patients (p < 0.001). On the other hand no difference was found in serum SGOT and serum SGPT (*p* = 0.092 and *p* = 0.514, respectively) levels suggesting that the elevation in ALP could not be atributed to abnormal liver function (Table 1). All bone markers, except magnesium, *were not associated* with *glycemic control* as measured by HBA1c (Table 2).

## Discussion

In the present study, serum RANKL levels were significantly elevated in T1D patients compared to controls. Since serum RANKL promotes osteoclastogenesis higher levels of serum RANKL might be associated with increased osteoclast activation. On the other hand, OPG, a decoy RANKL receptor, plasma levels were significantly lower in T1D patients compared to controls. Overall the significantly higher RANKL/OPG ratio in T1D patients may indicate increased osteoclast differentiation and activation, enhanced bone resorption leading to osteopenia and osteoporosis [11].

In the literature there are controversial results regarding OPG and RANKL circulating levels in T1D patients (Table 3) [14–18]. Our finding of significant lower OPG levels compared to controls, is in agreement with several studies, in which no microvascular complications of T1D were noted [14, 15]. On the contrary, elevated OPG levels have been positively associated with the progression of diabetes and the appearance of its complications [16, 19, 20]. Regarding age, a bimodal distribution of OPG levels with age is reported, with peak OPG levels during infancy and after 45 years of age [21, 22]. Although we found a significant negative association between OPG levels and age in the control group, this association was not observed in T1D patients. On the other hand, it is well established that parathyroid hormone and vitamin D increase RANKL gene expression (Fig. 2a), while vitamin D upregulates and PTH downregulates OPG expression (Fig. 2b) [23–25]. Interestingly there is a heterogeneity among the different studies regarding PTH and 25(OH) D vitamin levels. Therefore, different levels of PTH and 25(OH) D and the presence of diabetic complications could *explain* discrepancies in circulating *levels* of OPG and *RANKL* between different studies in children (Table 3).

**Table 3:** Studies investigating OPG and RANKL circulating levels in children with T1D

	OPG	RANKL	25(OH)D	PTH	DC
Singh et al. [14]	↓	n.s.	n.s.*	n.s.	no
Abd et al. [15]	↓				no
Galluzzi et al. [17]	↑		n.s.	n.s.	no
Tsentidis et al. [18]	↑	↑	n.s.	↓	no
Lambrinoudaki et al. [16]	n.s.	n.s.			↑cIMT

*n.s.* non significant, *DC* reported diabetic complications, *cIMT* carotid intimal thickness

* 1,25(OH) D was low in T1D group

Our study indicates that PTH and 25(OH) D may contribute significantly to the alterations in circulating concentrations of OPG and RANKL. Vitamin D is a steroid hormone essential for calcium homeostasis and bone remodeling [26]. Vitamin D deficiency is involved in the impairment of insulin synthesis and secretion and may be considered as an environmental factor with a pivotal role in the pathogenesis of T1D [27, 28]. In our study, serum 25(OH) D levels in T1D patients were significant lower compared to controls, in agreement with previous studies [29, 30]. Interestingly, duration of disease correlated with decreased 25(OH) D serum levels.

On the other hand, PTH acts on osteoclasts and stimulates osteolysis and bone resorption [31]. This osteoclastogenic effect is accomplished in part through the induction of RANKL and downregulation of OPG gene expression, respectively [32]. Serum PTH levels were significantly higher in T1D patients than in controls, in agreement with previous studies [29, 33]. It is well documented that serum Ca, P and Mg levels are stimuli for PTH secretion [31]. Interestingly, no significant difference was found in serum Ca, P and Mg levels between patients and controls. Therefore, one possible explanation for the PTH upregulation could be the low levels of 25(OH) D observed in our study [31, 34].

## Conclusion

Our study demonstrated that OPG and RANKL may be promising biomarkers for T1D patients. However, an understanding of what determines OPG and RANKL levels in T1D patients is not completely clear. Circulating levels of OPG and RANKL were associated with several factors including parathormone, vitamin D and therefore, may represent an integrative biomarker for a variety of endocrine signaling disturbances observed in T1D.

## Data Availability

The datasets analysed during the current study are available from the corresponding author on reasonable request.
